# Fuzzy PROMETHEE model for public transport mode choice analysis

**DOI:** 10.1007/s12530-023-09490-4

**Published:** 2023-02-18

**Authors:** Laila Oubahman, Szabolcs Duleba

**Affiliations:** 1 Department of Transport Technology and Economics, Faculty of Transportation and Vehicle Engineering, Budapest University of Technology and Economics, Műegyetem rkp. 3., Budapest, 1111 Hungary; 2 Institute of Mathematics and Informatics, University of Nyíregyháza, Sóstói u.31/b., Nyíregyháza, 4400 Hungary

**Keywords:** PROMETHEE, Fuzzy, MCDA, GAIA plane, Public transport, Mode choice

## Abstract

The importance of public transportation service quality research is significantly increasing in recent years, it is the key to understanding and analyzing passengers’ preferences. Different approaches are utilized to explore users’ preferences however, dominantly these apply merely subjective scoring of the attributes and alternatives of the mobility. In this paper, we design a specific model for public transportation mode choice which is capable of integrating subjective scoring with scoring by objective measures such as distance or time. Owing to this purpose, we combine the outranking Preference Ranking Organization METHod for Enrichment Evaluation (PROMETHEE) as a method to evaluate passengers’ preferences for tangible and intangible criteria with the fuzzy theory, and the Graphical Analysis for Interactive Aid (GAIA) plane to visualize the interactions between attributes as well as to test the robustness of the results via sensitivity analysis. The contribution of this paper is the constructed integrative method that is less subjective than the well-known models but also keeps the freedom of individual evaluators in expressing their preferences. Moreover, another significant issue of mode choice analysis is the group consideration, which is also refined in the new methodology by taking into account not only the mean of group preferences but also their range. A common characteristic of public surveys, the possible vague responses of the layman pattern is solved with the fuzzy approach to reduce the risk of uncertain scoring. The proposed model acts as a great base for the fuzzy inference system that can facilitate mode choice for passengers within a changing environment. The efficiency of the new methodology is demonstrated through a real-world case study of Budapest city, the obtained results are supporting underground mode service quality and highlighting its impact on citizens’ behavior in favor of public transport.

## Introduction

The public transport network is one of the critical domains for several countries (Alkharabsheh et al. 2021). Assuring the expected service quality is a challenge for municipalities, decision-makers, and service providers. In parallel with the aim of increasing ridership ratio (van Lierop and El-Geneidy 2016), policymakers strive to reduce the private mode use, because of the negative externalities causing pollution, congestion, and resource consumption (Beirão and Sarsfield Cabral 2007; Rotaris et al. 2022).

As the public transport network has huge importance in facilitating all citizens’ daily life, it is necessary to involve actual and potential users in the decision-making process, to explore, identify and meet their expectations (Fearnley et al. 2011). Passengers’ behavior is influenced both by objective and subjective factors, these factors differ from one user to another (Santos et al. 2013).

For instance, psychological factors, such as the feeling of safety and comfort inside the vehicle and in the stop station, have an important impact on mode choice preferences, this was proved by German, Swiss, Vietnamese and Canadian communities (Fujii and Van 2009; Scherer and Dziekan 2012; Legrain et al. 2015), the economic factor, as well as the travel time and the frequency of lines, are also influencing the public transportation mode choice for passengers (i.e. bus, tram, underground mode, walk, bike) (Bunschoten et al. 2013).

Recently, the consideration of mode choice preferences has become a target to ameliorate and expand services. Different approaches are adopted to come out with models and scenarios to upgrade public transport facilities. Using Multi-Criteria Decision Aid methods (MCDA); such as the Analytical Hierarchy Process (AHP), PROMETHEE, and the Technique for Order of Preference by Similarity to Ideal Solution (TOPSIS) (Kiciński and Solecka 2018; Alkharabsheh et al. 2021; Oubahman and Duleba 2021a; Chrysafis et al. 2022), and Discrete Mode Choice approach (Dell’Olio et al. 2011; Hasnine and Habib 2018) have demonstrated positive feedbacks both from the theoretical and the practical sides in constructing consensual models. We note that out of these techniques only PROMETHEE is capable of considering not only subjective scoring but also a more objective measure e.g. distance and time thresholds in the case of some criteria. Fuzzy approaches are also utilized to avoid uncertainty and vagueness of decision-makers (Aikhuele and Oluwadare 2019; Ebrahimi and Bridgelall 2020; Spiliotis et al. 2021). In statistical approaches, the characterization of a group is completed by not only calculating the mean but also adding standard deviation or any range measure of the data. Consequently, for the recent study, instead of the sole use of the arithmetic or geometric means to aggregate a group of evaluations, the upper and the lower bounds of responses can also be included in the analysis. Therefore, the combination of both approaches seems promising for creating an integrated and efficient model. The Fuzzy AHP method is widely used in literature because of its simplicity and effectiveness in process evaluation, especially in public transportation (Bilişik et al. 2013; Alkharabsheh and Duleba 2021; Çelikbilek et al. 2022). Furthermore, the fuzzy PROMETHEE approach has reached significant results to solve decision-making problems in life-threatening fields and with high precision, such as selecting a nuclear power site (Wu et al. 2020), medicine and healthcare (Bilsel et al. 2006; Ozsahin 2020), as well as environmental issues such as waste treatment studied by Lolli et al. (2016).

Our paper’s goal is to execute the Fuzzy PROMETHEE model to assess public transport services; especially for bus, tram, and underground modes. Intangible evaluations are common in the literature (Bernasconi et al. 2014; Coffey and Claudio 2021). However, this study considers tangible attributes as well, avoiding their subjective evaluation by a linguistic scale as all MCDM techniques but assigning exact values from decision-makers for sufficient evaluations. The targeted pattern is the public transportation users, thus the group of evaluators are not experts. Consequently, the risk of uncertainty of the responses due to lack of information or motivation of this layman pattern is high, so it is handled by the fuzzy theory to reduce bias in the scoring. To avoid the risk of overgeneralization that features the previous models, three categories are taken into account; the upper, the mean, and the lower values of scores. As a close connection to the field of evolving systems, the created model acts as a promising base for the fuzzy inference system that supports public transportation users to choose the optimal mode in an evolving environment such as; different prerequisites or different groups of decision-makers (Aikhuele and Oluwadare 2019).

The outranking PROMETHEE method eliminates scaling effects within attributes by utilizing six different preference functions (Brans et al. 1986; Le Téno and Mareschal 1998). The advantage of making the PROMETHEE method distinguished; is the cardinal output in a form of the GAIA plane (Geometric Analysis for Interactive Aid), this feature simplifies the understanding of the interaction between criteria. The direction of the optimum solution to the problem is designated by the decision axis and decision-maker brain visualization. It is illustrated as a circle around the decision axis. The attributes in the same direction and length with this axis have good performance, and the attributes pointing in opposite directions are called conflicting attributes. Sensitivity analysis is possible by changing the criteria’s weights when the direction of the decision axis may change while alternatives and criteria keep the same position (Kabir and Sumi 2014).

In previous research, the PROMETHEE method was utilized in different domains to select optimal solutions for the environmental, manufacturing, information technology, and transportation sectors (Wang and Yang 2006; Dağdeviren 2008; Roozbahani et al. 2012). This variety of applications explains the strength of the method and encourages its applicability in the transportation field as well via the use of integrated models and combinations with other MCDA methods. Combining PROMETHEE with the Fuzzy approach which was first introduced by Zadeh (1965) and extended for decision-making methods by Dijkman et al. (1983), enriches our model to cope with evaluations’ uncertainty, especially in the case of large-scale decision-maker evaluations. In literature, the number of evaluators in PROMETHEE models is generally low; for instance (Lolli et al. 2016) considered only three decision-makers, large-scale surveys are in their infancy in this methodology. Elevli (2014) and Tong et al. (2020) evaluated the actions based on the assessments of five decision-makers. However, in our study, large-scale entries (with 100 completed questionnaires) from evaluators are collected to construct the Fuzzy-PROMETHEE model, exploiting the GAIA plane tool to visualize the cardinal results.

The contributions of the recent paper can be summarized as follows:

- to reduce the problem of subjective scoring in public transport mode choice by adding measures to some variables.
- to deal with the group preferences in a more sophisticated way by paying attention to the range of scoring.
- to apply a large-scale pattern in a PROMETHEE model for acquiring preferences of a wider community to make the final conclusion more reliable.
- to mitigate the risk of untrustworthy scoring of the civil evaluators.
- to ensure the evolving approach to react to the changes in the transport environment.

In further sections, the literature review is introduced in Sect. 2, the methodology is in Sect. 3. Results and discussion of the case study of Budapest city in Sect. 4, followed by the paper’s main conclusions.

## Literature review

Due to the fact that public transportation mode choice preferences change according to passengers’ constraints (Nutsugbodo et al. 2018), it is crucial to provide such level of service quality that meets passengers’ expectations (Çelikbilek et al. 2022). Various research aimed to identify the key solution to improve public transport’s supply quality and to increase ridership ratio together with reducing road congestion, pollution, and fuel consumption (Soltanpour et al. 2018). For instance, (Redman et al. 2012) identified quality attributes that attract private mode users. (Ebrahimi and Bridgelall 2021) determined factors that impact the attraction of public transportation mode choice. Moreover, Gruyter et al. (2021) expressed the importance of the distance to stop factor in the use of public transportation modes. Many research have studied only subjective aspects for attributes to solve a decision-making process (Escobar and Moreno-Jiménez 2007; Nalmpantis et al. 2019; Amenta et al. 2021). However, in our model, we separate measurable from non-measurable attributes, and we give exact numbers to these measurable variables for the PROMETHEE entries.

Furthermore, in the scientific literature, the majority of the models adopt the conventional approaches for aggregating individual preferences such as the geometric or the arithmetic means to conclude a group decision (Blagojevic et al. 2016; Oubahman and Duleba 2022). This causes overgeneralization of the scoring without knowing the range of evaluations and the dispersal information between the best and the worst points is lost. In our model, we tried to fill this gap by embedding lower and upper bounds to gain a more sophisticated image of the involved pattern.

For non-expert evaluators, the scoring uncertainty of the collected data may result in unreliable final decisions. To reduce the risk of untrustworthy evaluations, the fuzzy theory is used in this study to overcome the ambiguity and the divergence of the data in the decision-making process, especially when it is related to collecting evaluations from different decision-makers that are not experts and might have insufficient motivation or information for scoring.

There are some previous examples for preference models applying fuzzy PROMETHEE theory. The authors Ayadi et al. (2021), Ziemba (2021), Bilişik et al. (2013) created a hybrid fuzzy methodology to measure customer satisfaction in public transport network in Istanbul city. Tong et al. (2020) combined the fuzzy theory with PROMETHEE method to create a consensual model serving industry field. Another study by Ghasemi and Talebi (2014) utilized Fuzzy PROMETHEE model with GAIA analysis to construct a group decision support system. Moreover, Celik et al. (2013) introduced an integrated model of fuzzy MCDM methods to ameliorate public transport customers’ satisfaction. A summary of the existing MCDA and Fuzzy theory studies is presented in Table 1.

**Table 1:** Literature review’s summary

References	MCDM method	Methodology
Shahmardan and Hendijani Zadeh (2013)	Entropy Fuzzy-Fuzzy PROMETHEE	Combination of Fuzzy theory with PROMETHEE method for tangible and intangible aspects for MCDM problem
Geldermann et al. (2000)	Fuzzy PROMETHEE	Fuzzy PROMETHEE model for the assessment of the environmental policies
Goumas and Lygerou (2000)	Fuzzy PROMETHEE	Fuzzy PROMETHEE approach for reliable outranking results
Moslem et al. (2019)	Fuzzy AHP-interval AHP	Fuzzy theory and AHP approach for sustainable transport development decision
Celik et al. (2013)	Fuzzy AHP-Fuzzy TOPSIS	Hybrid model to evaluate customer satisfaction regarding public transportation in Istanbul city
Kiciński and Solecka (2018)	AHP–ELECTRE	A comprehensive evaluation of urban transport in Krakow using a hybrid AHP-ELECTRE model
Nalmpantis et al. (2019)	AHP	AHP method for the evaluation of innovative ideas for urban transport
Oubahman and Duleba (2022)	AHP-PROMETHEE	A comparative study between AIJ and AIP approaches for the aggregation of a group evaluations using the AHP-Group PROMETHEE model
Bergqvist et al. (2015)	MAMCA	Multi Actor Multi Criteria Analysis model to improve transportation sustainability by evaluating four potential measures
Qi et al. (2021)	Discrete choice model	Discrete choice model to predict and improve long-term travel time to facilitate decision making process
Anagnostopoulos et al. (2003)	PROMETHEE-GAIA	Evaluation of transportation infrastructure
Whalen et al. (2013)	Discrete choice model	Factors affecting mode choice preferences for university students
Oubahman and Duleba (2021a)	Group PROMETHEE	Evaluation of urban public transport by using PROMETHEE methos

Based on the thorough literature review some research gaps could be identified and our paper’s aim is to partially or completely fill these gaps.

- most of the public transport preference surveys apply merely a linguistic scale that makes the evaluations very subjective.
- group characterization is overgeneralized by using means for aggregating the individual scores without paying attention to the range of scoring.
- the evolving issue is not sufficiently considered even if the transport system environment might change over time.
- uncertainty of scoring, especially for citizens or layman participants is not properly handled.

## Methodology

### PROMETEE method

PROMETHEE is a Multi-Criteria Decision Aid (MCDA) method that evaluates alternatives based on pre-defined criteria, it was introduced by Brans in 1982 and extended by Brans and Vincke in 1986 (Brans et al. 1986). The first phase of the PROMETHEE method is to define criteria to maximize and the ones to minimize, followed by selecting the suitable preference functions. It is worth mentioning that different preference functions can be chosen in the same model (Oubahman and Duleba 2021b).

Therefore, defining PROMETHEE thresholds enables each decision-maker to set their preferences for each criterion. After the calculation of positive and negative flows, the PROMETHEE partial ranking is provided without any loss of information. In case of incomparability, the comprehensive ranking via PROMETHEE II has proceeded. PROMETHEE provides cardinal output GAIA that facilitates the understanding of the interactions between criteria vis alternatives and visualizes the optimal solutions (Macharis et al. 1998; Christian et al. 2016).

Considering a set of criteria $C=\left\{{\text{g}}_{1},\dots .,{\text{g}}_{\text{m}}\right\}$ and a set of alternatives $A=\left\{{a}_{1},\dots .,{a}_{n}\right\}$. The pairwise comparison and the amplitude of deviation $d$ between two alternatives ${a}_{i}$ and ${a}_{{i}^{\prime}}$ with $\left\{i\cdot{{i}^{\prime}}\right\}\in \left\{1,\dots .,n\right\}$ and $i\ne {{i}^{\prime}}$ for ${g}_{j}$ criterion, $j=\left\{1,\dots .,m\right\}$ is calculated, as shown in Eq. (1)

1 $${d}_{j}\left({a}_{i},{a}_{{i}^{\prime}}\right)={g}_{j}\left({a}_{i}\right)-{g}_{j}\left({a}_{{i}^{\prime}}\right)$$

In this paper, we used two preference functions; ‘Usual criterion’ and ‘Quasi-Criterion’. ‘Usual criterion’ is utilized for qualitative (i.e. non-measurable evaluation) criteria with the evaluation of 5-point scale (very bad, bad, average, good and very good) as the case of safety of stop and comfort in stop. While quasi-criterion is the most suitable for quantitative (i.e. measurable evaluation) criteria by the reason of the indifference threshold that eases the decision process (i.e. for distance of stop criterion, if the difference between two stops is 500 m the passenger chooses the closest). The characteristics of the selected functions are explained in Eqs. (2)–(3).

Type I: usual criterion

2 $$P(d) = \left\{\begin{array}{ll}0 \quad if\;d\le 0\\ 1 \quad if\;d>1\end{array}\right.$$

Type II: quasi-criterion

3 $$P(d) = \left\{\begin{array}{ll}0\quad if\;d\le q\\ 1 \quad if \; d>q\end{array}\right.$$

$q$ is the indifference threshold defined by the decision-maker, $P()$ is the preference function chosen based on criterion’s characteristics to compute the preference between two alternatives.

The computation of the positive flow ${\phi }^{+}\left({a}_{i}\right)$; which measures how the alternative ${a}_{i}$ is outranking the other alternatives, and the negative flow ${\phi }^{-}\left({a}_{i}\right)$; that evaluates how other alternatives are outranking the alternative ${a}_{i}$ through PROMETHEE I is the next step.

After assigning positive weights to $m$ criteria $\{{w}_{1},\dots .{w}_{m}\}$ with $\sum _{j=1}^{m}{w}_{j}=1$, the preference value $\pi$ can be calculated.

For $$\left\{{a}_{i},{a}_{{i}^{{\prime }}}\right\}\in A$$

4 $$\pi \left({a}_{i},{a}_{{i}^{\prime }}\right)=\sum _{j=1}^{m}{P}_{j}\left({a}_{i},{a}_{{i}^{\prime }}\right).{w}_{j}$$

The positive flow ${\phi }^{+}:$

5 $${\phi }^{+}\left({a}_{i}\right)= \frac{1}{n-1} \sum _{{a}_{{i}^{\prime }} \in A-\left\{{a}_{i}\right\}}\pi ({a}_{i},{a}_{{i}^{\prime }})$$

The negative flow ${\phi }^{-}:$

6 $${\phi }^{-}\left({a}_{i}\right)= \frac{1}{n-1} \sum _{{a}_{i} \in A-\left\{{a}_{{i}^{\prime }}\right\}}\pi ({a}_{{i}^{\prime}},{a}_{i})$$

Three judgments can be concluded between each two alternatives from the partial ranking (PROMETHEE I). It can be a preference relation (P), an indifference relation (I), or incomparability (R). In every case, some conditions should be fulfilled. Please see Appendix Eq. (21).

PROMETHEE II comes to overcome the incomparability identified in PROMETHEE I, it equals the subtraction of the negative flow ${\phi }^{-}$ from the positive flow ${\phi }^{+}$.

7 $${\Phi } \left({a}_{i}\right)= {\phi }^{+}\left({a}_{i}\right)- {\phi }^{-}\left({a}_{i}\right)$$

Alternative preference increases with the value of the net flow $\Phi$ which reveals two assumptions: preference and indifference.

Preference (P): $${a}_{i}{P}^{II}{a}_{{i}^{\prime }}$$

8 $${\Phi} \left({a}_{i}\right) > {\Phi} \left({a}_{{i}^{{\prime}}}\right)$$

Indifference (I): $${a}_{i}{I}^{II}{a}_{{i}^{\prime }}$$

9 $${\Phi} \left({a}_{i}\right) = {\Phi} \left({a}_{{i}^{{\prime}}}\right)$$

### GAIA plane

Cardinal visualization is also possible for the PROMETHEE method. GAIA plane enables the understanding of the interaction between attributes. The decision axis designates the direction of the alternatives and the criteria that are better performing compared to the ones in the opposite directions. Thus, the positive interaction is recognized between two attributes if they are in the same direction. Otherwise, a negative interaction is performed (Brans and Mareschal 1994).

### Fuzzy group PROMETHEE

The fuzzy PROMETHEE approach is adopted in this study to overcome the divergence of the evaluations, the approach is summarized in 6 steps, that are highlighted in the remarkable work of Lolli et al. (2016).

- Step 1: Weights assignment to criteria.
  Since the preference of the criteria is not always the same, we can consider $M$ decision-makers assigning weights $w$ to $m$ criteria $\left\{{g}_{1},\dots .,{g}_{m}\right\}$, the normalization of the weights is necessary in a way that the sum of the weights equals to 1, Eq. (10)
  10 $$\sum_{j=1}^{m}{w}_{j}=1$$
  The mean value does not take into account the dispersion of the judgments. Hence, a triangular fuzzy number is achieved for each criterion by including the lower and the upper values as explained in Eq. (11)
  11 $${\tilde{w}}_{j}=\left(l{w}_{j}, m{w}_{j} \cdot u{w}_{j}\right)$$
  ${l{w}_{j}}, {m{w}_{j}} \cdot {u{w}_{j}}$ are respectively, the lowest, the mean and the upper values of the set of weights $\left\{{w}_{1,j}, {w}_{2,j}, \ldots .,{w}_{M,j}\right\}$. Please see Appendix Eq. (22).
- Step 2 : Fuzzy decision matrix.
  After the expression of PROMETHEE scores by all decision-makers, the fuzzy scores for alternatives ${a}_{i}$ in the case of qualitative criteria are calculated as shown in Eqs. (12). Please see Appendix Eq. (23).
  12 $${ \tilde{x}}_{ij}= \left(l{x}_{i,j}, m{x}_{i,j} \cdot u{x}_{i,j}\right)$$
- Step 3: Fuzzy indifference and preference thresholds.
  With the same concept adopted for weights and alternative scores, the fuzzification of indifference and preference thresholds is necessary to cope with the divergence of decision-makers’ opinions.
  For indifference thresholds
  13 $${\tilde{q}}_{j}= \left(l{q}_{j}, m{q}_{j}. u{q}_{j}\right)$$
  For detailed equation, please see Appendix Eq. (24).
  For preference thresholds
  14 $${\widetilde{ p}}_{j}= \left(l{p}_{j}, m{p}_{j} \cdot u{p}_{j}\right)$$
  For detailed equation, please see Appendix Eq. (25).
- Step 4: Fuzzy preference function.
  The fuzzification of preference functions between two alternatives ${x}_{i \cdot j}$ and ${x}_{{i}^{{\prime}},j}$ comes as a result of previous fuzzification, and it is computed as; Eq. (15).
  15 $$\tilde{P}({x}_{i,j}, {x}_{{i}^{{\prime}},j})= \left(l{P}_{i,{i}^{{\prime}},j}, m{P}_{i{i}^{{\prime}},j}. u{P}_{i{i}^{{\prime}},j}\right)$$
  For detailed equation, please see Appendix Eq. (26).
- Step 5: Fuzzy positive, negative, and net flows.
  Similarly, we calculate the fuzzy flows considering the same concept, Eqs. (16)–(18) show the formulas used in the study.
  Leaving flow
  16 $${ \tilde{\varphi }}_{i}^{+} = (l{\varphi }_{i}^{+}, m{\varphi }_{i}^{+} , u{\varphi }_{i}^{+})$$
  For detailed equation, please see Appendix Eq. (27).
  Entering flow
  17 $${ \tilde{\varphi }}_{i}^{-}= (l{\varphi }_{i}^{-}, m{\varphi }_{i}^{-} , u{\varphi }_{i}^{-})$$
  For detailed equation, please see Appendix Eq. (28).
  Net flow
  18 $${\tilde{\Phi }}_{i}= ( {l\Phi }_{i}, {m\Phi }_{i}, u{\Phi }_{i} )$$
  For detailed equation, please see Appendix Eq. (29).
- Step 6: Defuzzification.
  Defuzzification is made to rank the alternatives based on the value of one indicator, instead of conflicting results of the two boundaries and the mean. It is highlighting the preferences allocated to each alternative. Different defuzzification methods can be used,, the centroid method, the mean-max membership, the center of sums, the max-membership principle, in this paper we adopt the approach presented by Tong et al. (2020) in order to gain a persuasive ranking.
  19 $${\Phi }_{i} = ({l\Phi }_{i},+ 4* {m\Phi }_{i}+ u{\Phi }_{i})/6$$
  To summarize the proposed methodology, Fig. 1 demonstrates the main steps.
  **Fig. 1:**
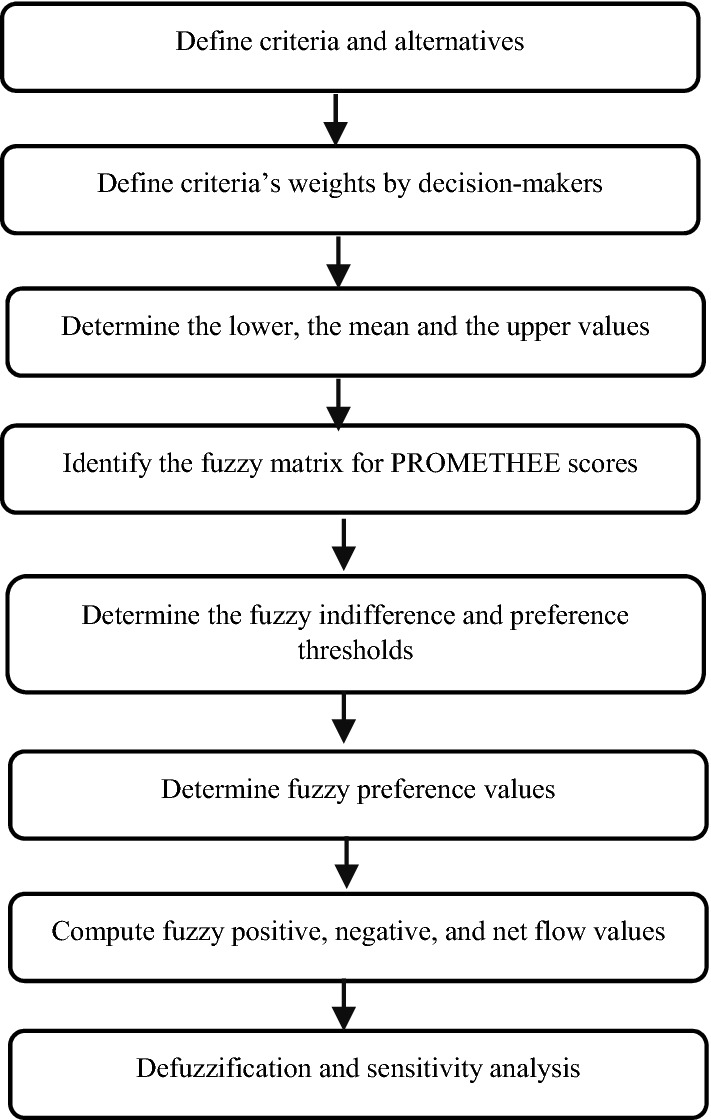
The description of the utilized methodology

## Results and discussion

We execute the presented methodology to evaluate public transport service quality in the city of Budapest. The chosen attributes for the evaluations are the following ten decision elements; distance to stop, comfort in stop, safety of stop, need to transfer, fit connection, frequency of lines, limited time of use, journey time, awaiting time, and time to reach stops. The explanation of these attributes is presented in Table 2. 100 evaluators, all of them are using public transport networks on a daily basis have been involved in the survey. Three transport modes are evaluated in this study; bus, tram, and underground modes, as they are the main travel alternatives to use in the examined city for daily travel.

**Table 2:** The explanation of evaluating attributes adopted in the model (Duleba 2022)

Criteria	Interpretation
Distance to stop—C1	Proximity of origin stations
Safety of stop—C2	Subjective feeling
Comfort in stop—C3	Seats, cooling system, heating system
Need to transfer—C4	Need to change the vehicle to reach the destination
Fit connection—C5	Time connection between lines to reach the destination
Frequency of lines—C6	Frequency of buses, trams, and underground modes
Limited time of use—C7	Time between the first and the last line of a day
Journey time—C8	The time between on-board and getting off from the vehicle
Awaiting time—C9	Waiting time in the station for the line
Time to reach stop—C10	Time to reach the origin station

The conduction of the comprehensive survey targeted the daily basis passengers, to analyze their assessments of public transportation. 100 passengers participated in this evaluation. Statistically, the number of the samples is not representative, but the MCDA approach provides a profound perception of the study based on pairwise comparisons better than a simple survey (Saaty 1977) due to considering merely the sufficiently consistent evaluations that mitigate the risk of bias in the results. The survey was conducted in November and December 2020, and the average time to fill out the complete survey was 20 min. The first section includes evaluators’ general information. The second section assesses the objective and subjective values for PROMETHEE by considering the detailed level of the structure containing 10 criteria, while the last one concerns the socio-demographical characteristics. The participants are from different ranges of age 50% are between 18 and 25 years old, 30% are from the range 26–40 years old, and 20% are over 41 years old. 49% of the respondents are males and 51% are females. These characteristics correspond to the public transport user population of Budapest with a slight underrepresentation of the older citizens. The single tickets are used by a total of 10% and 90% are using monthly passes.

Evidently, the quality of the service provided by each mode is not the same. Passengers’ preferences change in the course of the day depending on different factors; thus, we aim the assessment these preferences deeply to define the key motivations in the selection.

We consider that the criteria are having equal weights for simplification purposes, therefore, all the weights are equal to one. $\forall j\in \left(1,\dots .m\right),\forall M\in ({DM}_{1}, \ldots .{DM}_{k})$

20 $${w}_{M,j}=1$$

$M$ is the number of decision-makers, $j$ is the criterion

Defining cost and benefit criteria is a mandatory phase in PROMETHEE models. In this study, there are seven cost criteria; distance to stop, need to transfer, fit connection, frequency of lines, journey time, awaiting time and time to reach stop, these criteria should be minimized as much possible as it can be. On the other hand, benefit criteria are only three; safety of stop, comfort in stop, and limited time of use, which have to be maximized.

The chosen preference function is a quasi-criterion function for all criteria except for the safety of stop, comfort in stop and limited time of use that are assumed to be evaluated using the usual criterion preference function.

Table 2 defines all of the applied ten criteria in the model.

**Fig. 2:**
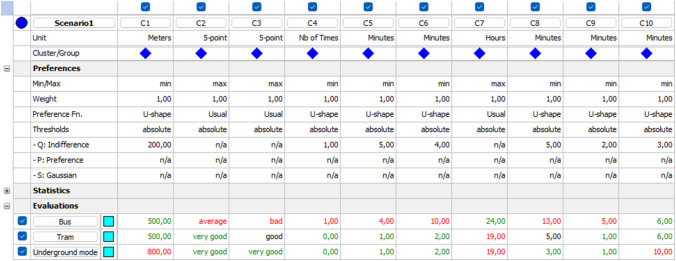
An example of PROMETHEE entries for one evaluator

Figure 2 shows an example of PROMETHEE entries for an arbitrary evaluator participating in the surveying process. As previously was highlighted, exact values are assigned to certain alternatives according to objective criteria referring to the evaluator’s own experience for daily travel. Additionally, indifference thresholds are defined to proceed with preference function computations.

Out of the ten attributes, merely two, Safety of stop and Comfort in stop have been evaluated by a subjective Likert-scale, in all other cases, we applied objective measures as meters or minutes. We emphasize that this is still not an observation-based analysis (so total objectivity cannot be assumed) but the risk of subjective scoring is mitigated by providing the evaluators anchors of objective measures to select the perceived scores. This is a real benefit compared to both of the widely applied methodology groups, DCM and MCDM type surveys and techniques.

Following the presented methodology, the fuzzy decision matrix, the fuzzy indifference and preference thresholds, the fuzzy preference function, and the fuzzy flows that are respectively explained in step 2, step 3, step 4, and step 5 are adopted in our case study to evaluate the passengers’ evaluations, the lower, the mean and the upper values are identified for each criterion with reference to the alternatives and indifference thresholds.

It is clear that this study will result in three sub-sections in step 6, which are the lower flow values, the mean flow values, and the upper flow values. It is worth mentioning that flow computation has been made by using the Visual PROMETHEE software (Promethee 2013).

### Lower values

The first outputs to be analyzed are the lower values. Apparently, as it is shown in Table 3, considering these values for both partial and complete ranking that are representing the outputs of PROMETHEE I and PROMETHEE II, the underground mode is the most preferred mode followed by tram and bus modes. No incomparability is detected.

**Table 3:** Positive, negative, and net flows for lower values

Lower values	${\text{l}{\varphi }}^{+}$	${\text{l}{\varphi }}^{-}$	$\text{l}{\Phi }$	Partial ranking	Complete ranking
Bus mode	0.1	0.2	− 0.1	3	3
Tram mode	0.1	0.15	− 0.05	2	2
Underground mode	0.25	0.1	0.15	1	1

**Fig. 3:**
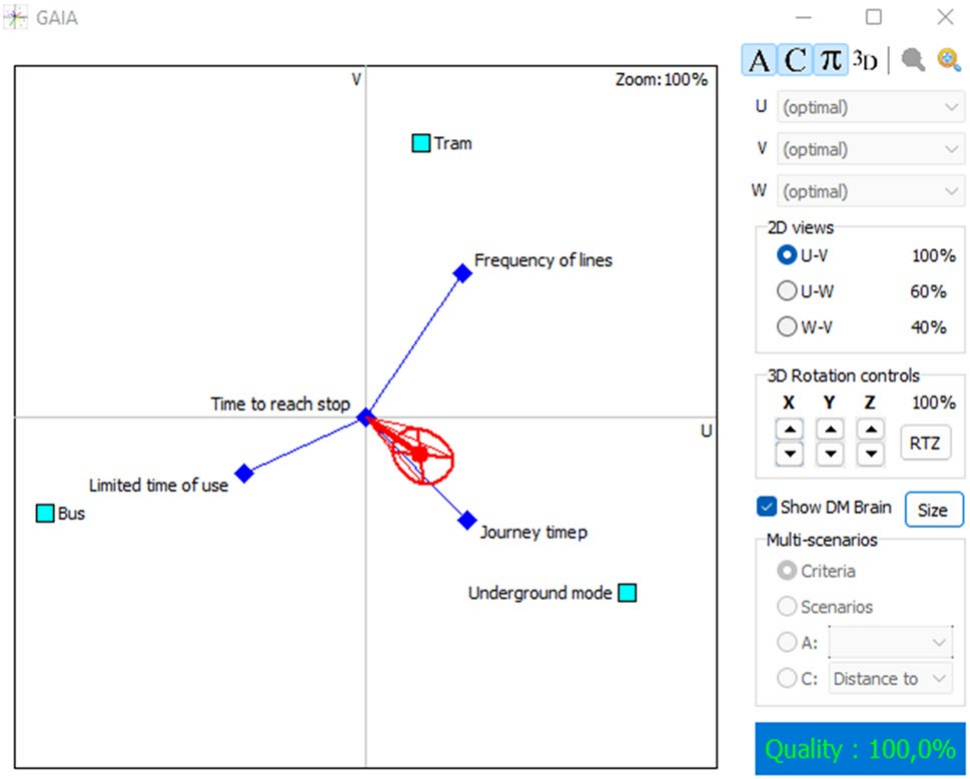
GAIA plane 2D for lower values

**Fig. 4:**
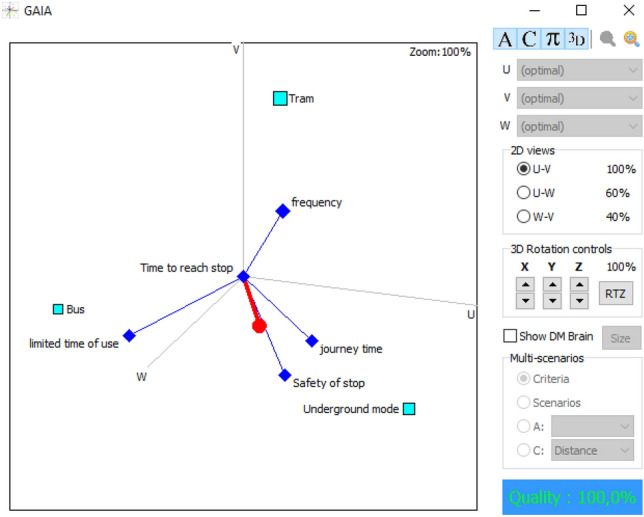
GAIA plane 3D for lower values

Exploiting the advantages of PROMETHEE for the cardinal outputs, the GAIA plane from visual PROMETHEE software provides a sight of the interaction between criteria and alternatives. Figures 3 and 4, show how the attributes are performing. The red decision axis and the red circle around the axis are illustrating the decision-makers’ brains. The axis is pointing in the direction of the optimal alternative, in this case; underground mode is in the same direction as the decision axis, which means that it performs very well, especially with respect to journey time and safety of stop. On the other hand, tram mode and frequency of lines are pointing in the same direction, confirming the positive interaction. The same is between bus mode and limited time of use, which is evident because buses operate 24 h per day which is not the case for the other modes. Other criteria have neutral judgments because of the determined values of the thresholds.

**Fig. 5:**
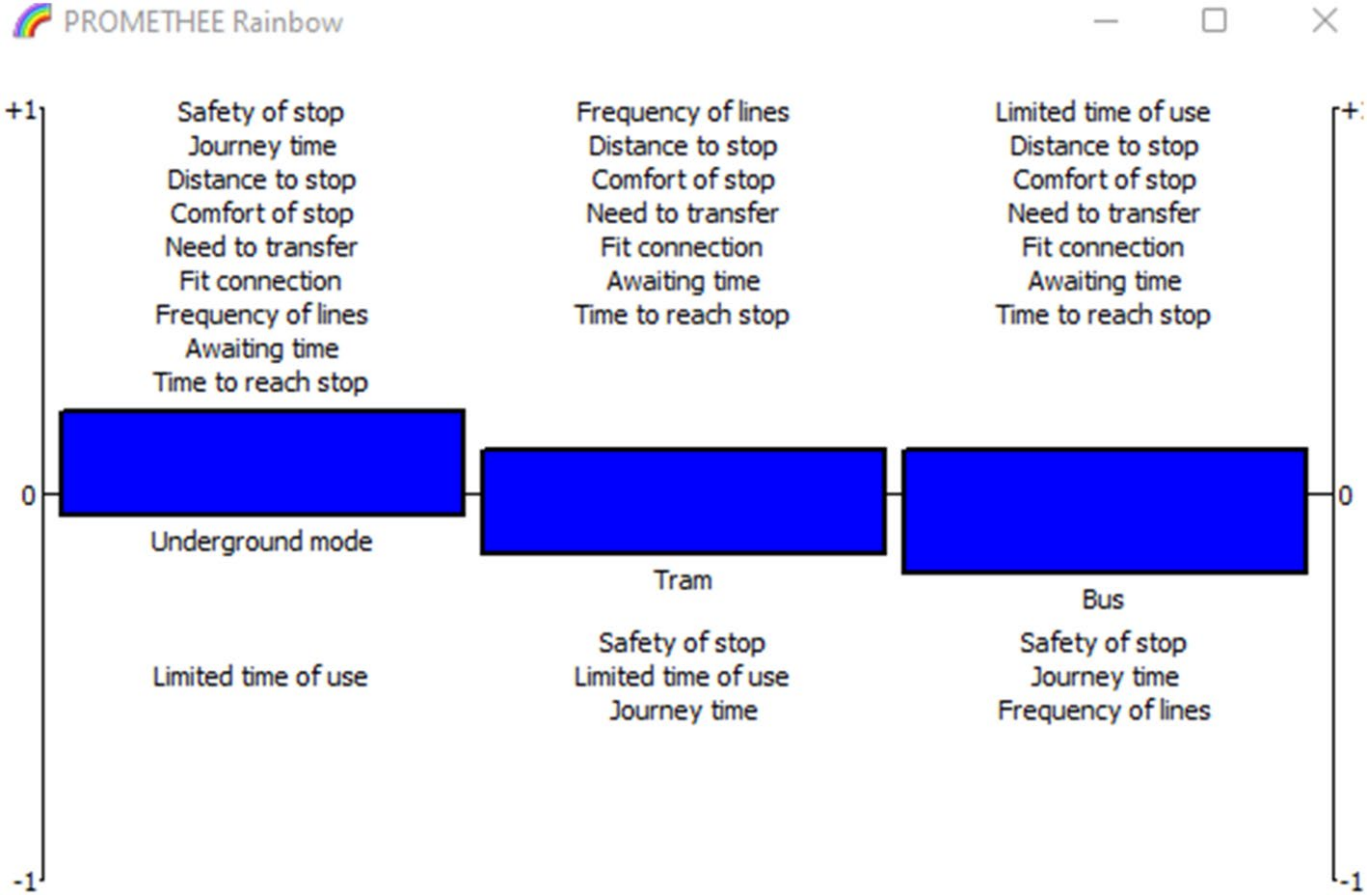
PROMETHEE rainbow for lower values

For better visibility of the performance of the alternatives according to criteria, PROMETHEE Rainbow is introduced in Fig. 5. The alternatives are ranked in increasing order from the left to the right. For underground mode, all the criteria are placed in the interval [0, 1] except the limited of time criterion, which is placed in the negative section [− 1, 0[. The positive section is the highest compared to other alternatives and the negative section is the smallest. For tram mode, it has only three criteria in the negative section [1, 0[. Safety of stop, limited time of use and journey time. The rest of the criteria have positive evaluations. Bus mode has also three criteria with low evaluations that are safety of stop, journey time and frequency of lines.

### Mean values

**Fig. 6:**
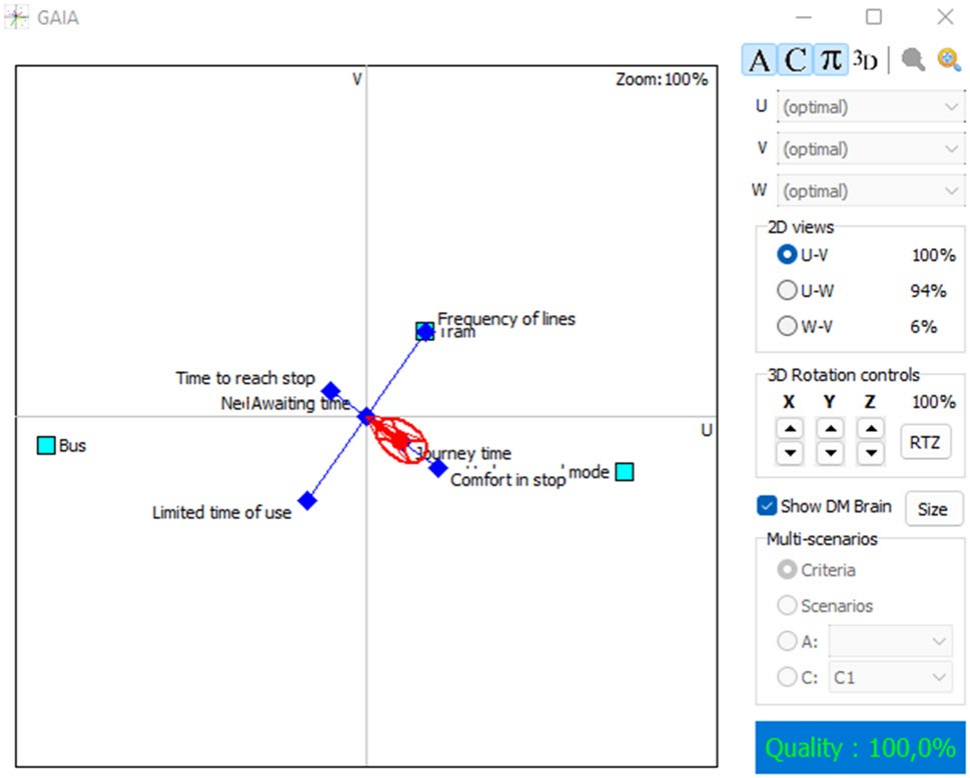
GAIA plane 2D for mean values

**Fig. 7:**
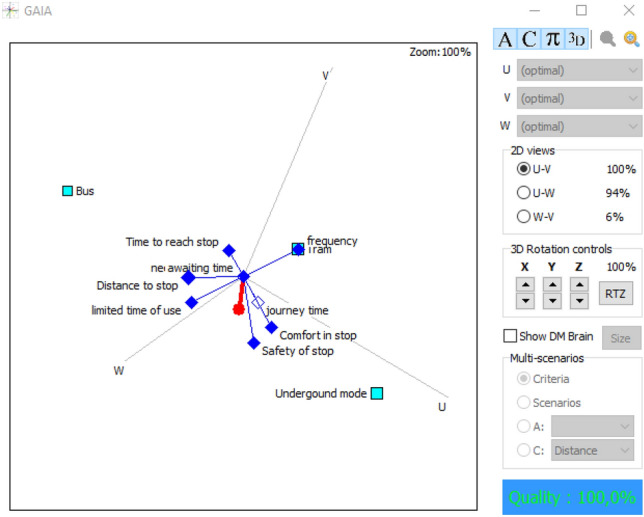
GAIA plane 3D for mean values

Similarly, the values of the flows computed in PROMETHEE I and PROMETHEE II considering the mean values are presented in Table 4. The underground mode has the highest value for the positive flow and the lowest value for the negative flow. Thus, it is ranked in the first position in the PROMETHEE I. An incomparability relation is detected between bus and tram modes, hence, proceeding to the complete ranking through PROMETHHEE II is necessary to solve this incomparability. The complete ranking revealed that the second position goes to tram mode, while the bus is in the last position. This ranking is supporting the results of the lower values ranking. Attributes’ interactions in Figs. 6 and 7, disclose the significant performance of underground mode, which is close to the decision-makers brain, in parallel with a positive interaction with the safety of stop, comfort in stop, and journey time that are pointing in the same direction and adjoining the decision axis. Tram is close to the decision axis compared to bus, this explains its ranking in the 2nd position, which is compact with the frequency of lines attribute, shedding light on the remarkable performance. The bus is in the opposite direction of the decision axis, three attributes are pointing in its direction; distance to stop, limited time of use, and time to reach stops. This visualization is reflecting reality; because bus stops are omnipresent in household areas, with a huge number of lines compared to other modes, and operates 24 h per day.

**Table 4:** Positive, negative, and net flows for mean values

Mean values	${\text{m}{\varphi }}^{+}$	${\text{m}{\varphi }}^{-}$	$\text{m}{\Phi }$	Partial ranking	Complete ranking
Bus mode	0.2	0.35	− 0.15	Incomparability	3
Tram mode	0.15	0.15	0		2
Underground mode	0.3	0.15	0.15	1	1

**Fig. 8:**
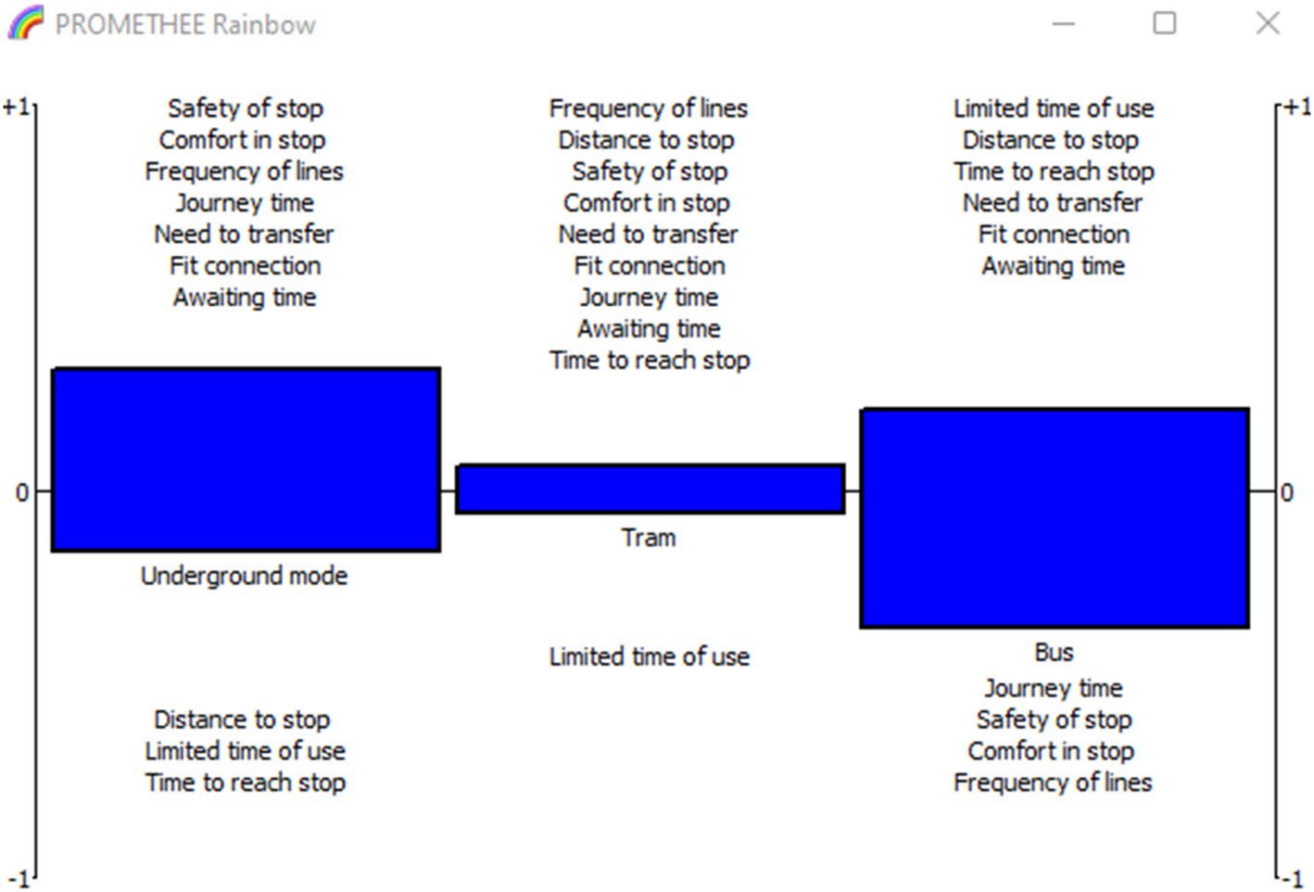
PROMETHEE rainbow for mean values

PROMETHEE Rainbow in Fig. 8, shows the performance of the alternatives regarding the criteria, the upper section [0, 1] is for the good evaluation, however, the interval [− 1, 0] is for the weak evaluation.

### Upper values

Identically, in this section, we focus on upper values assigned by decision-makers, the PROMETHEE I and PROMETHEE II result in the same ranking. The bus is ranked in the first position, followed by underground mode, then tram mode in the last position. These results are expected and logical, because of the proximity of the bus to the households and the number of lines that can be considered huge compared to tram and underground modes (Table 5).

**Table 5:** Positive, negative, and net flows for upper values

Upper values	${\text{u}{\varphi }}^{+}$	${\text{u}{\varphi }}^{-}$	$\text{u}{\Phi }$	Partial ranking	Complete ranking
Bus mode	0.25	0	0.25	1	1
Tram mode	0	0.2	− 0.2	3	3
Underground mode	0.05	0.1	− 0.05	2	2

**Fig. 9:**
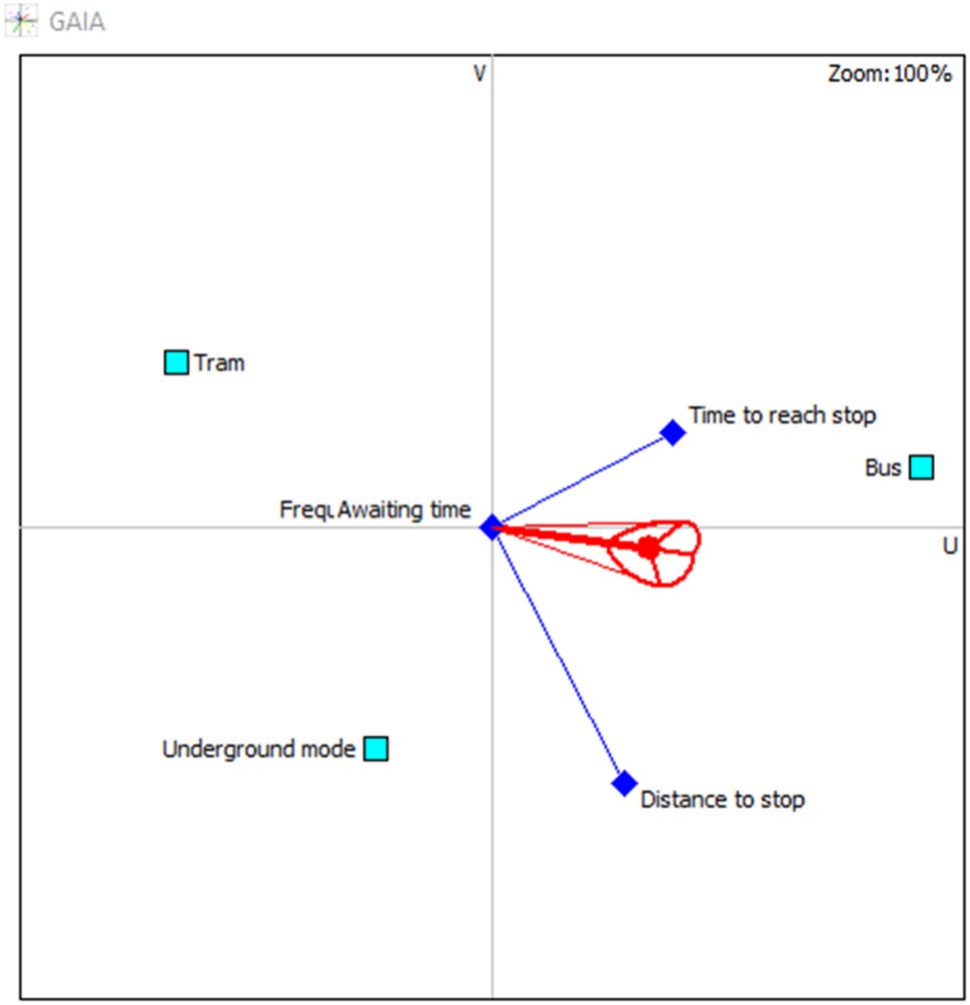
GAIA plane 2D for upper values

**Fig. 10:**
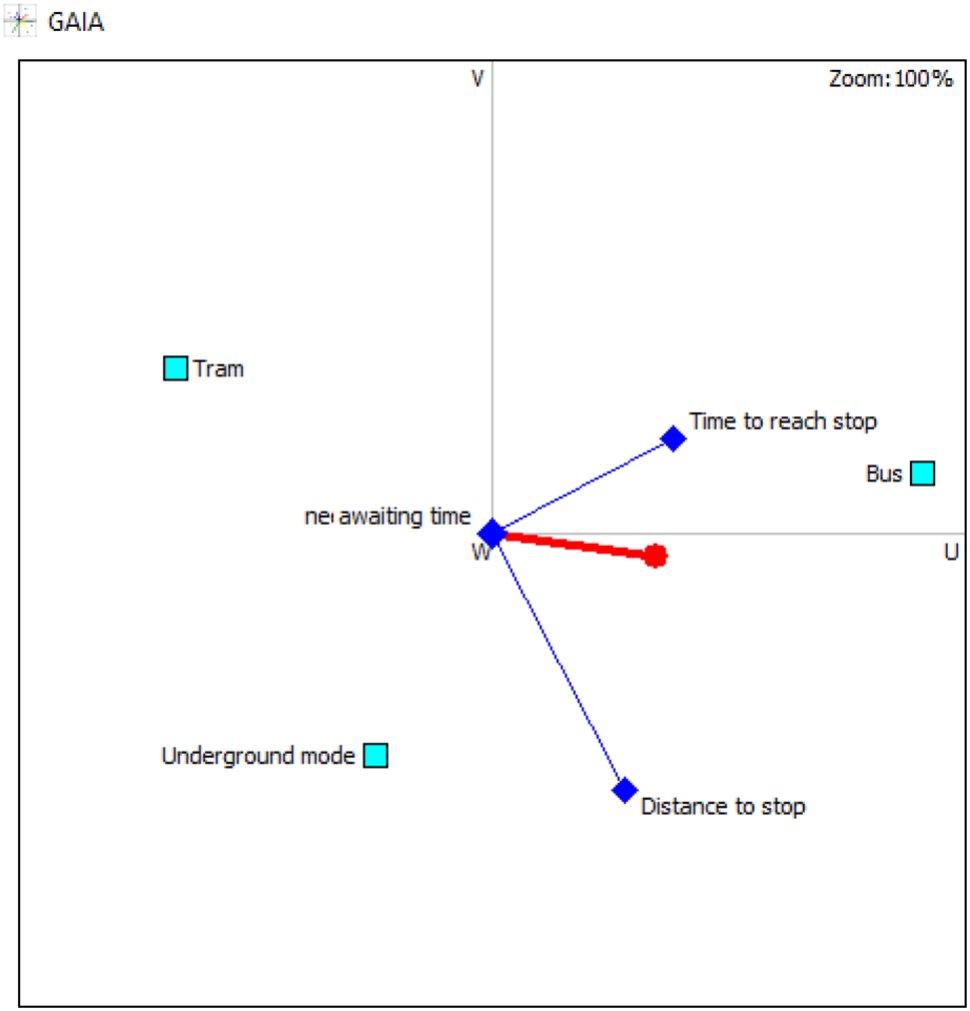
GAIA plane 3D for mean values

Evidently from Figs. 9 and 10, bus mode is the most preferable, it is in the same direction as the decision axis and decision-makers brain illustrations and interacts positively with distance to stop and time to reach stop attributes. Other criteria are neutral in this judgment, they are all placed at the intersection of the axes (U–V). Tram and underground modes are situated in the opposite direction, as a result of the low performance. However, the underground mode is close to the direction of the decision axis compared to the tram, which is the reason for its second-ranking position.

It is worth highlighting that all GAIA plane visualizations are showing the quality of the views as 100%, affirming the non-loss of any information with the cardinal visualization. This is considered a great advantage in visualizing these results.

**Fig. 11:**
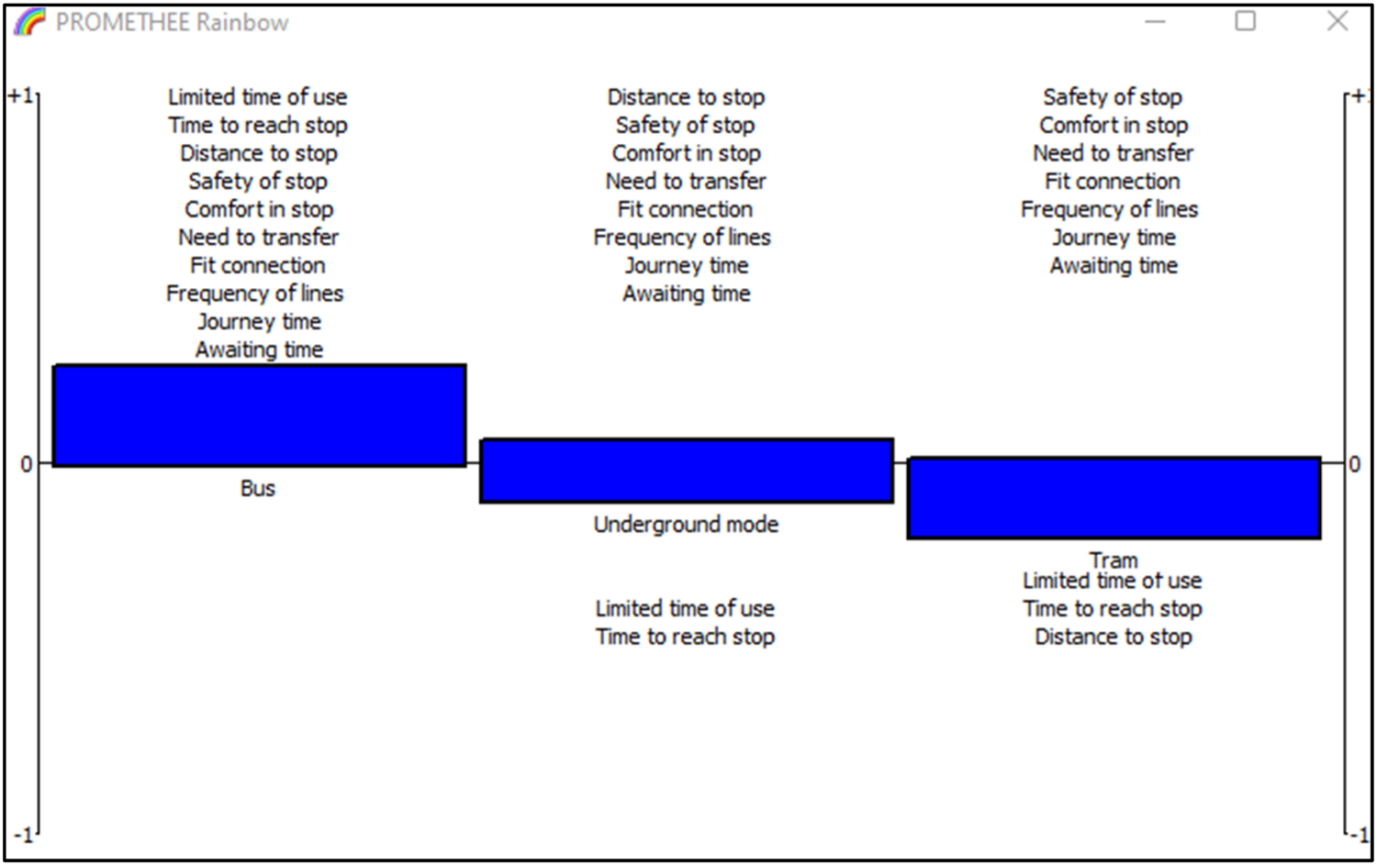
PROMETHEE rainbow for upper values

Similarly, the PROMETHEE rainbow Fig. 11 demonstrates the ranking of alternatives. All the criteria are placed in the upper section for bus mode, underground mode has weak performance in limited time of use and time to reach stop. While tram has three criteria with weak performance.

### Defuzzification

According to step 6, we apply Eq. (19), for the defuzzification of the results. The final ranking of the alternatives stated in Table 6, is as follows; the underground mode is ranked in the first position, reflecting its great importance in influencing mode choice with reference to the evaluated criteria, it performs in both comfort in stop, the safety of stop, journey time, awaiting time. On the other hand, the tram is positioned in the second place, this mode is distinguished by the frequency of its lines. The last mode is bus mode, thanks to the numerous bus stops located in the examined city, it performs uniquely in distance to stop, time to reach stops, and limited time of use since it operates 24 h per day.

As shown in Table 6, the incomparability relation is highlighted in PROMETHEE I between bus and tram modes. It is coped with PROMETHEE II that outranked tram on bus mode, spotting light on the importance and the preference of the tram mode by passengers over the bus.

**Table 6:** Positive, negative, and net flows after defuzzification

Defuzzification	${{\varphi }}^{+}$	${{\varphi }}^{-}$	${\Phi }$	Partial ranking	Complete ranking
Bus mode	0.191667	0.266667	− 0.075	Incomparability	3
Tram mode	0.116667	0.158333	− 0.04167		2
Underground mode	0.25	0.133333	0.116667	1	1

This paper considers passengers’ mode choice preferences specifically within public transportation modes to analyze the service quality distinction between public transportation modes. To validate our results and demonstrate the effectiveness of the proposed model, we compare the findings with other previous studies aiming for the same objective of mode choice preferences. To the best of our knowledge, there is no official and detailed data about the usage of public transportation modes in Budapest city. However, the authors Puhe and Schippl (2014) introduced a study about urban transportation, and emphasized that 47% of the participants in the survey use public transportation in Budapest city, and 20% prefer their private cars, 32% of the participants choose walking and 1% use bike. The participants stated the importance of comfort and travel time as the most influencing factors in their mode choice. This assumption for the two factors is aligned with our results, stating the high evaluation of underground mode on different criteria and especially for travel time and comfort.

Bounded transportation such as tram and underground mode, are the most preferred modes by the participants in our study, rail factor’s impact has been proven by previous studies. Passengers’ attitude in Krakow, a central Europe city with the same similarities as Budapest city, is influenced by the rail factor and the surveyed community preferred tram mode (Kiciński and Solecka 2018), Swiss and German communities emphasized the rail factor (i.e. tram, underground mode) on their mode choice (Scherer and Dziekan 2012), this clearly demonstrates the effectiveness of the constructed model. Note that our proposed methodology can be easily implemented in an application supporting the mode choice decision of the applier. For this, actual input data of preferences are necessary that reflect changes in the certain transportation system. Thus, the evolving characteristic of this model could be assured by refreshing some data on the public transport system (stop-reallocation, new schedule, etc.) and the current preferences of the user. Moreover the individual preferences might be compared to community preferences over time that might help in making more sustainable mode choices. An overview of research supporting our results is summarized in Table 7,

**Table 7:** Comparison of the applied methodology in this paper and previous studies

References	The objective	Targeted pattern	Results	Methodology
Beirão and Sarsfield Cabral (2007)	Understanding citizens’ attitude toward private cars and public transportation	Community of Porto—Portugal	– Citizens’ behavior is influenced by service level of transport system – Citizens are dependent on their private modes	Qualitative study
Scherer and Dziekan (2012)	Psychological rail factor on mode choice	German and Swiss communities	– 75% of Swiss community and 63% of German community are psychologically influenced by rail factor	Statistical approach
Kiciński and Solecka (2018)	Analyze scenarios with possible public transport connections	Community of Krakow—Poland	– Tram connections were identified as the best alternatives for Krakow’s community	MCDA-AHP and ELECTRE
Alkharabsheh and Duleba (2021)	Public transportation service quality evaluation during Covid-19	Community of Amman—Jordan	– Passengers choice depends on Journey time and frequency of lines to choose bus lines	MCDA-Fuzzy AHP and Kendall Model
Limanond et al. (2011)	Mode choice preferences inside university campuses (Private car–bus–bicycle)	Students of the campus of Suranaree university of technology—Thailand	– Car ownership is a major element influencing mode choice – Lack of cycling infrastructure and moderate bus service decrease the attractiveness of these modes	Qualitative approach
Proposed study	Analysis of mode choice preferences within public transportation including the three available public transportation modes	Community of Budapest—Hungary	– Reduce the problem of subjective scoring in public transport mode choice by adding measures to some variables – Deal with the group preferences in a more sophisticated way by paying attention to the range of scoring – Apply a large-scale pattern in a PROMETHEE model for acquiring preferences of a wider community to make the final conclusion more reliable – Mitigate the risk of untrustworthy scoring of the civil evaluators – Ensure the evolving approach to react to the changes in the transport environment	MCDA– Fuzzy PROMETHEE

### Sensitivity analysis

Sensitivity analysis examines the robustness of the results. Visual PROMETHEE provides this feature via the walking weights interface (Promethee 2013). We tested alternatives ranking by changing the criteria’s weights for the three categories.

**Fig. 12:**
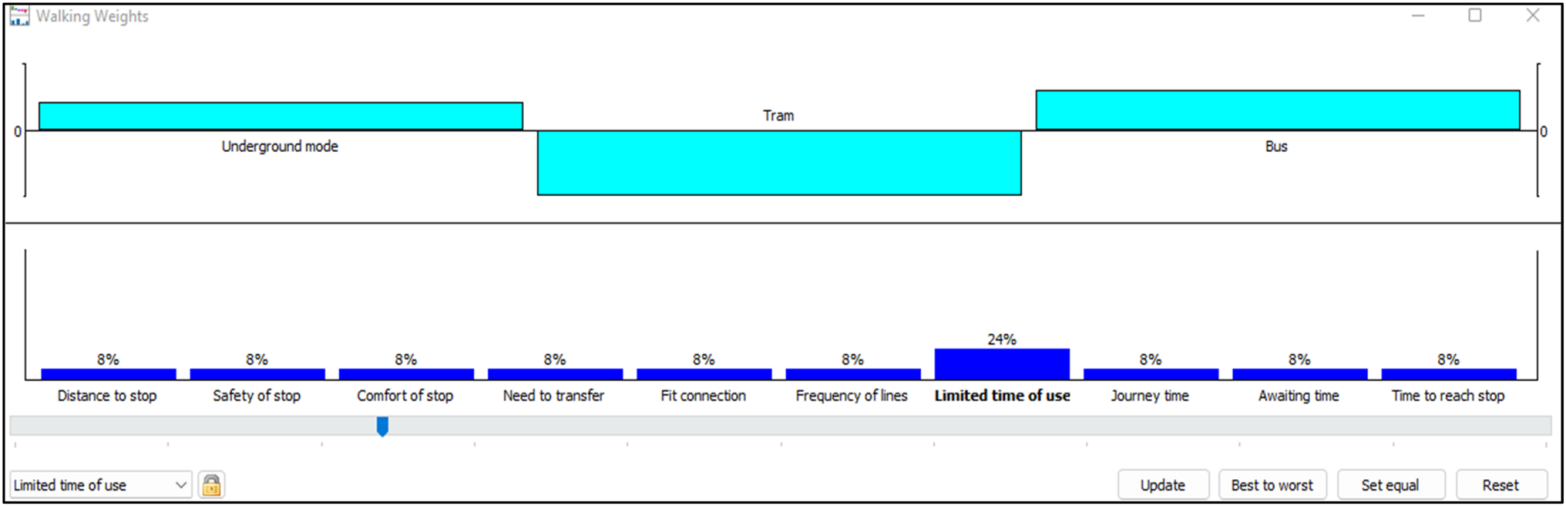
Walking weights for lower values

For the lower values, the ranking is changed by increasing the weight of limited time of use to 0.24, this criterion was selected because it has positive values for only bus mode according to the PROMETHEE Rainbow Fig. 5. The remaining criteria are equally weighted 0.08. The bus mode became in the first position followed by underground and tram modes (please see Fig. 12).

**Fig. 13:**
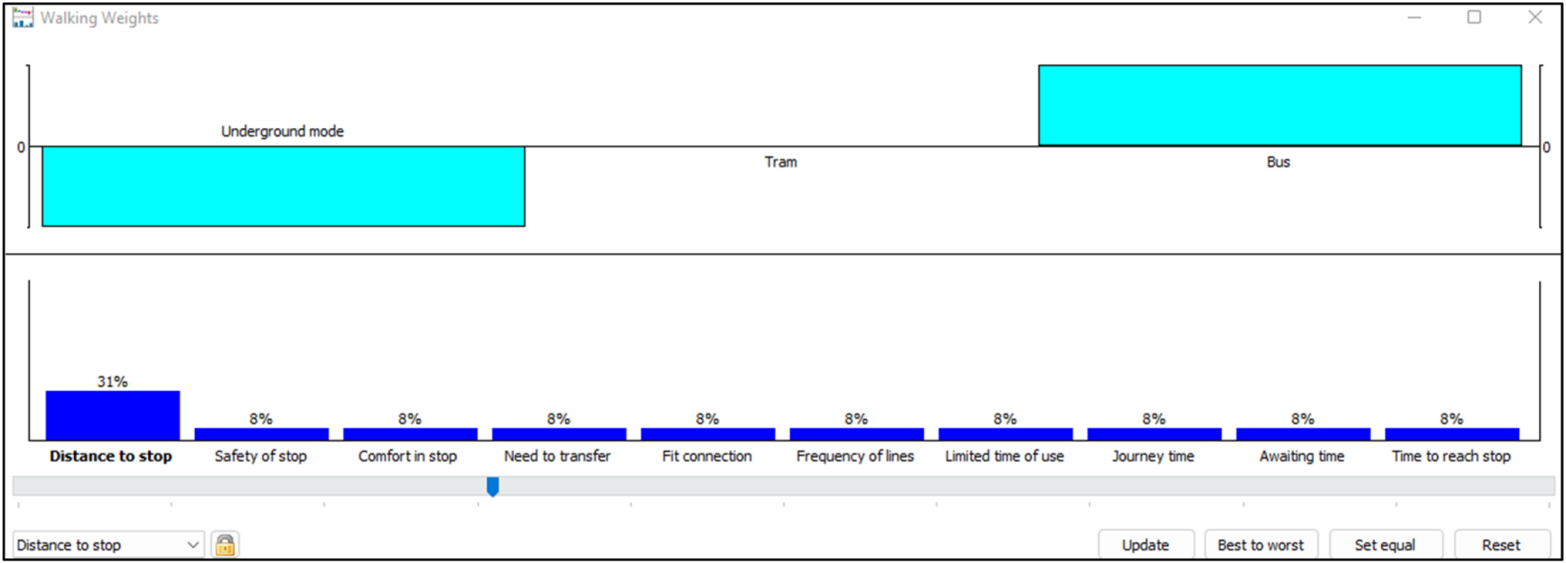
Walking weights for mean values

For mean values Fig. 13, the chosen criterion is distance to stop, when its weight reaches the value of 0.31, the ranking has changed: bus becomes in the first position, followed by tram mode and then underground mode.

**Fig. 14:**
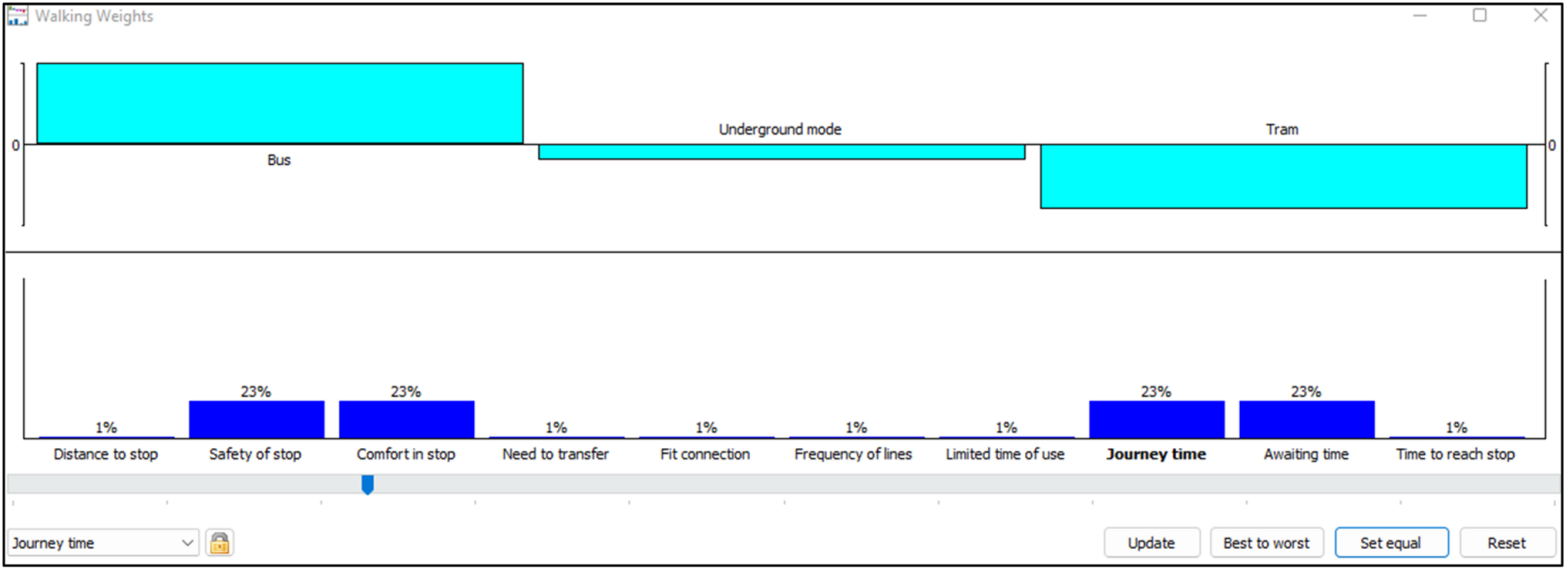
Walking weights for upper values

Identically, the same reasoning is applied to the upper values. According to the PROMETHEE rainbow (Fig. 11), all the criteria are placed in the positive section for bus mode, this explains the non-change of the ranking after modifying the weights for four criteria safety of stop, comfort in stop, journey time, and awaiting time to 0.23 (Fig. 14). The ranking cannot be changed because of the advantages of bus mode for upper values.

## Conclusion

This paper aims to present mode choice preferences within the public transport network by handling tangible and intangible variables simultaneously and considering group heterogeneity more sufficiently. In group decision-making, instead of the mean values only, the upper and the lower values were included in the analysis. Due to the layman pattern, the PROMETHEE method has been combined with the fuzzy approach to reduce the uncertainty of the scoring. The model has been applied successfully for the case of Budapest city. In the model, altogether ten criteria were defined to evaluate three different public transport modes. The computation of the PROMETHEE I and PROMETHEE II indicators went through the fuzzification and defuzzification process. The model is also strengthened by the cardinal output (GAIA plane) that visualizes the positive and negative interactions between attributes and assists the selection of the optimum action in a minimal time for analysis, thanks to the PROMETHEE rainbow, walking weights and sensitivity analysis.

The obtained results are demonstrating the impact of service quality on passengers’ choices. Underground mode is significantly winning first place, expressing the best preference for the passengers, followed by tram mode and bus mode in the third position. We can also conclude from the GAIA representations that bus mode is well performing in distance to stop and time to reach stop criteria, whereas it is omnipresent in household areas, which is evident, since it does not require a huge budget to construct specific roads or stop stations as well as the possibility of operating 24 h per day, which is not the case of the rail modes (tram, underground mode). These advantages make bus lines management easier, and policymakers may focus on increasing the quality of the service of that mode, especially for comfort in stop, the safety of stop, frequency of lines, journey time, and so on. Tram and underground modes are having a good service quality according to the pre-defined criteria, the biggest obstacle facing decision-makers in adding new lines to attract more people; is the needed financing to construct new lines for new destinations, the quality of the service provided is significant for underground mode, especially in the side of comfort, safety, and journey time. Tram is favorable in terms of attracting passengers to use that mode. These results are reflecting the strategy adopted by policymakers, hence, taking into consideration these findings in new projects might impact positively the attitude of the community toward public transportation modes, such as targeting the amelioration of bus service quality with reduced journey time and frequency of line and improved safety and comfort in stops.

This paper, to the best of our knowledge, is the first study combining the Fuzzy PROMETHEE together with GAIA plane in the public transportation field’s assessment. However, it proves powerful results that are supporting the study made by the authors of Oubahman and Duleba (2021a). This approach does not require much time or resources to evaluate all the records separately, instead of an immense number of calculations, only three cases can be proceed; the lower, the mean, and the upper values. The model can be executed repetitively in the case of any changes in public transportation services, attributes or in the case of different groups of evaluators such as; passengers, potential passengers, policymakers, or municipality representatives, the model is capable of gaining the ranking of the existing modes. It represents a great base for a Fuzzy Inference System (FIS), which can provide passengers the opportunity to introduce their prerequisites to identify the optimum public transport mode.

All of our objectives could be completed as demonstrated in the previous section. Subjective scoring could be reduced by implementing some measures in the evaluation process (e.g. distance to stop 500 m).

The authors of recent paper encourage the application of this approach in other research to reduce time and effort together with exploiting cardinal results to facilitate the decision-making process along with reduced subjectivity compared to other mainstream models.

As for the limitations of our study, only the opinion of public transport users to outrank the three modes was considered. Other stakeholders’ evaluations can support the comparison of the evaluations from different perspectives. Furthermore, other modes can be included in future studies such as cycling mode to compare preferences of all available modes in the presence of other factors, in particular weather, suitable infrastructure, and fare values.

## Data Availability

The datasets used and/or analyzed during the current study are available from the corresponding author on reasonable request.

## Appendix

21 $$\begin{aligned} & {\text{Preference}}\;(\text{P}){:} \; {a}_{i}{P}^{I}{a}_{i{\prime }} \quad \left\{\begin{array}{c}{ \varphi }^{+}\left({a}_{i}\right) > {\varphi }^{+}\left({a}_{{i}^{{\prime }}}\right) \; and \;{\varphi }^{-}\left({a}_{i}\right) < {\varphi }^{-}\left({a}_{{i}^{{\prime }}}\right)\\ { \varphi }^{+}\left({a}_{i}\right) > {\varphi }^{+}\left({a}_{{i}^{{\prime }}}\right) \;and \; {\varphi }^{-}\left({a}_{i}\right) = {\varphi }^{-}\left({a}_{{i}^{{\prime }}}\right) \\ {\varphi }^{+}\left({a}_{i}\right) = {\varphi }^{+}\left({a}_{{i}^{{\prime }}}\right) \; and \; {\varphi }^{-}\left({a}_{i}\right) < {\varphi }^{-}\left({a}_{{i}^{{\prime }}}\right)\end{array}\right. \\ & {\text{Indifference}} \; (\text{I}){:} \; {a}_{i}{I}^{I}{a}_{i{\prime }}\quad {\varphi }^{+}\left({a}_{i}\right) = {\varphi }^{+}\left({a}_{{i}^{{\prime }}}\right) \; and \; {\varphi }^{-}\left({a}_{i}\right) = {\varphi }^{-}\left({a}_{{i}^{{\prime }}}\right) \\ & {\text{Incomparability}} \; (\text{R}){:} \; {a}_{i}{R}^{I}{a}_{i{\prime }} \quad \left\{\begin{array}{c}{ \varphi }^{+}\left({a}_{i}\right) < {\varphi }^{+}\left({a}_{{i}^{{\prime }}}\right) and {\varphi }^{-}\left({a}_{i}\right) > {\varphi }^{-}\left({a}_{{i}^{{\prime }}}\right)\\ { \varphi }^{+}\left({a}_{i}\right) >{ \varphi }^{+}\left({a}_{{i}^{{\prime }}}\right) \; and \; {\varphi }^{-}\left({a}_{i}\right) < {\varphi }^{-}\left({a}_{{i}^{{\prime }}}\right)\end{array}\right. \end {aligned}$$

22 $${\tilde{w}}_{j}= \left({min}_{m=1,.,M} \left\{{w}_{m,j}\right\}, \frac{1}{M} \sum _{m=1}^{M}{w}_{m,j}, {max}_{m=1,.,M} \left\{{w}_{m,j}\right\}\right)$$

23 $${ \tilde{x}}_{i,j}= \left({min}_{m=1,.,M} \left\{{x}_{m,i,j}\right\}, \frac{1}{M} \sum _{m=1}^{M}{x}_{m,i,j}, {max}_{m=1,.,M} \left\{{x}_{m,i,j}\right\}\right)$$

24 $${\tilde{q}}_{j}=\left({min}_{m=1,.,M} {q}_{m,j}, \frac{1}{M} \sum _{m=1}^{M}{q}_{m,j} , {max}_{m=1,.,M} {q}_{m,j}\right) \quad \forall j\in J$$

25 $${ \tilde{p}}_{j}=\left({min}_{m=1,.,M} {p}_{m,j}, \frac{1}{M} \sum _{m=1}^{M}{p}_{m,j} , {max}_{m=1,.,M} {p}_{m,j}\right) \quad \forall j\in J$$

26 $$\begin{aligned} l{P}_{i{,i}^{{\prime}},j} & = \left\{\begin{array}{c}1 \quad if \; min \left\{\right(u{x}_{i,j} - l{x}_{i{\prime },j}); (l{x}_{i,j} - u{x}_{i{\prime },j}\left)\right\} \ge u{p}_{j} \\ 0 \quad if \; min \left\{\right(u{x}_{i,j} - l{x}_{i{\prime },j}); (l{x}_{i,j} - u{x}_{i{\prime },j}\left)\right\} < u{p}_{j} \end{array}\right. \\ u{P}_{i{,i}^{{\prime }},j} & = \left\{\begin{array}{c}1 \quad if \; max \left\{\right(u{x}_{i,j} - l{x}_{i{\prime },j}); (l{x}_{i,j} - u{x}_{i{\prime },j}\left)\right\} \ge l{p}_{j} \\ 0 \quad if \; max \left\{\right(u{x}_{i,j} - l{x}_{i{\prime },j}); (l{x}_{i,j} - u{x}_{i{\prime },j}\left)\right\} < l{p}_{j} \end{array}\right. \\ m{P}_{i{,i}^{{\prime }},j} & = \left\{\begin{array}{c}1 \quad if \; m{x}_{i,j} - m{x}_{i{\prime },j} \ge m{p}_{j} \\ 0 \quad if \; m{x}_{i,j} - m{x}_{i{\prime },j} < m{p}_{j} \end{array}\right. \end{aligned}$$

27 $${ \tilde{\varphi }}_{i}^{+} = \left( \sum _{i= 1}^{n}\frac{\sum _{j=1}^{J}{lw}_{j}*{lP}_{{ii}^{\prime }}}{n-1}, \sum _{i= 1}^{n}\frac{\sum _{j=1}^{J}{mw}_{j}*{mP}_{{ii}^{\prime }}}{n-1}, \sum _{i= 1}^{n}\frac{\sum _{j=1}^{J}{uw}_{j}*{uP}_{{ii}^{\prime }}}{n-1} \right)$$

28 $${ \tilde{\varphi }}_{i}^{-} = \left( \sum _{i= 1}^{n}\frac{\sum _{j=1}^{J}{lw}_{j}*{lP}_{{i}^{\prime }i}}{n-1}, \sum _{i= 1}^{n}\frac{\sum _{j=1}^{J}{mw}_{j}*{mP}_{{i}^{\prime }i}}{n-1}, \sum _{i= 1}^{n}\frac{\sum _{j=1}^{J}{uw}_{j}*{uP}_{{i}^{\prime }i}}{n-1} \right)$$

29 $${{\tilde{\Phi }}_{i} =(l\varphi }_{i}^{+}- {l\varphi }_{i}^{-}, {m\varphi }_{i}^{+} - {m\varphi }_{i}^{-}, {u\varphi }_{i}^{+} - {u\varphi }_{i}^{-})$$
